# Time Course Evaluation of Enzyme-Linked Immunosorbent Assays Based on Cell-Free Recombinant Proteins for Detection of Antibodies against Middle East Respiratory Syndrome Coronavirus

**DOI:** 10.1128/spectrum.02953-22

**Published:** 2022-11-23

**Authors:** Jun Won Kim, Woo-Jung Park, Sung Soon Kim, Joo-Yeon Lee, Jeong-Sun Yang

**Affiliations:** a Korea Disease Control and Prevention Agency, Cheongju, South Korea; Quest Diagnostics

**Keywords:** Middle East respiratory syndrome, serological tests, coronavirus

## LETTER

Middle East respiratory syndrome coronavirus (MERS-CoV) infection has a high case fatality rate (1). Serological tests are used for patient management, seroprevalence surveys, passive immunotherapeutic studies, vaccine development, and identification of cases of asymptomatic infection. Enzyme-linked immunosorbent assays (ELISAs) based on recombinant MERS-CoV spike protein 1 (S1 protein) and nucleocapsid (N) protein are used to screen for MERS-CoV infection before confirmatory testing using neutralization assays (www.who.int/publications/i/item/10665-259952) (2, 3) but have not been fully validated for the time course of performance.

We developed and evaluated indirect IgG ELISAs using recombinant MERS-CoV N and S1 proteins (Kor/KNIH/002_05_2015) produced using a mammalian cell lysate-based cell-free protein expression system. This approach has several advantages over traditional ELISAs, including a shorter production process, the possibility of expressing multiple proteins simultaneously using PCR products without cloning and transformation, and provision of posttranslational modifications (4).

In this study, approved by the institutional review board of the Korea National Institute of Health (2016-05-08-P-A), a total of 205 serum samples, comprising a panel of 105 samples from 75 patients with confirmed MERS-CoV infection collected 1 to 171 days post–onset of the illness (DPI) and 100 samples from healthy MERS-CoV–negative controls, were tested. Preliminary cutoff optical density (OD) values of 0.43 and 0.46 were found for the negative-control samples, using the cell-free N and S1 IgG ELISAs, respectively. Using receiver operating characteristic curves, cutoff ODs of 1.1 and 1.2 were determined for the cell-free N and S1 IgG ELISAs, with sensitivities of 93.55% and 92.39%, respectively, specificities of 100%, and areas under the curve of 0.972 and 0.977, respectively.

**FIG 1:**
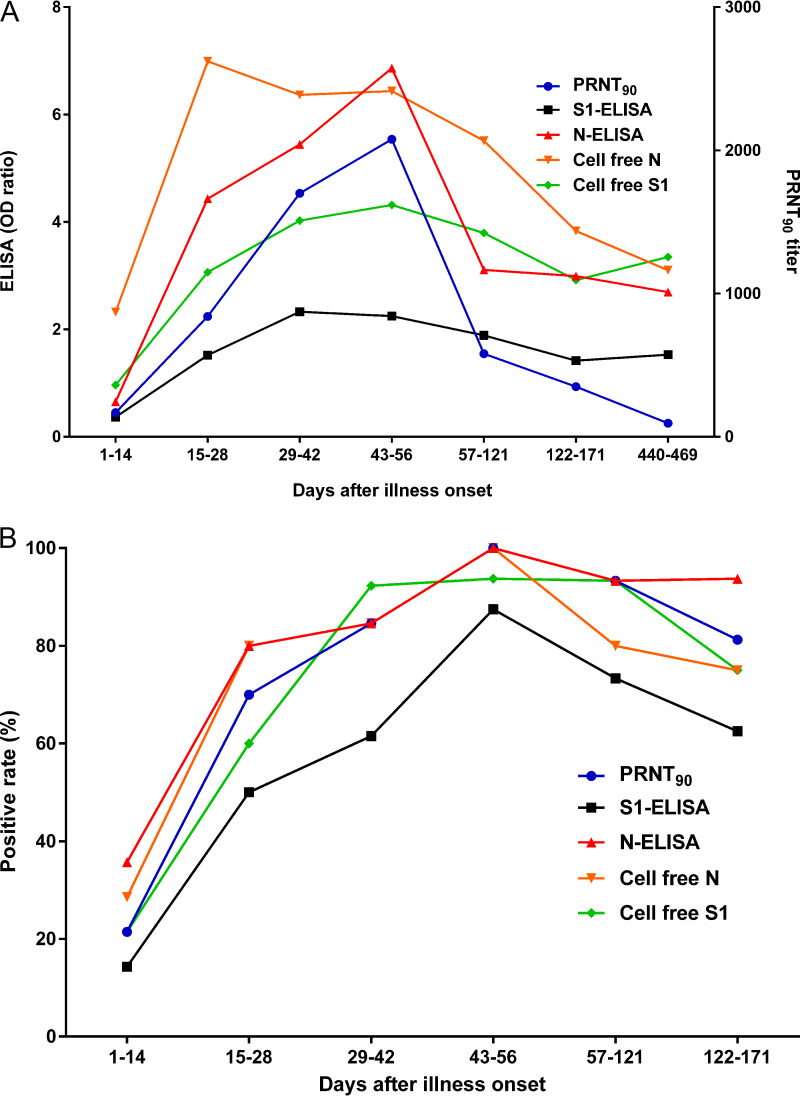
(A) Detection of anti-MERS-CoV IgG antibodies using ELISAs based on recombinant S1, N, cell-free S1, cell-free N proteins, and PRNTs on 105 serum samples collected from patients with MERS-CoV infection during an outbreak in South Korea. (B) Kinetics of IgG antibody responses in sera of patients with MERS-CoV infection. The OD ratio value for MERS-CoV S1 and N proteins were measured using S1- and cell-free S1-based IgG ELISAs, and N- and cell-free N-based IgG ELISAs, respectively. The neutralizing antibody titers were determined by PRNT_90_, and values of >20 were considered positive. The antibody OD ratio value or titer determined by each assay were compared based on the median value according to the days post–illness onset. ELISA, enzyme-linked immunosorbent assay; MERS-CoV, Middle East respiratory syndrome coronavirus; N, MERS-CoV nucleocapsid; PRNT, plaque reduction neutralization test; PRNT_90_, 90% plaque reduction neutralization test; S1, MERS-CoV spike 1.

We investigated the MERS-CoV IgG antibody response profiles and sensitivity of each assay by testing the 105 serum samples of MERS patients using cell-free N and S1 IgG ELISAs, commercial S1 (IgG) and N (IgG) ELISAs (EUROIMMUN AG, Lübeck, Germany, and Alpha Diagnostic International Inc., San Antonio, TX, USA, respectively), and plaque reduction neutralization tests (PRNTs) (Fig. 1). The assay sensitivity differed according to the DPI: the cell-free and commercial N ELISAs were the most sensitive 1 to 28 DPI; the cell-free S1 ELISA was the most sensitive 29 to 42 DPI; and the cell-free N ELISA and PRNT were the most sensitive 43 to 56 DPI. The cell-free N ELISA had the highest overall OD ratio value, and the cell-free N and S1 ELISAs had higher sensitivities than the commercial S1 ELISA and PRNT in most periods.

A testing algorithm involving the use of more than one assay may improve the diagnostic accuracy (5, 6). The N protein of coronavirus is abundantly expressed and highly immunogenic for eliciting antibody responses (7). Detectable N-specific antibody responses precede S-specific responses (8–10). Although N ELISAs have higher cross-reactivity than S1 ELISAs (6, 11), they may prevent underestimation of the MERS prevalence in seroprevalence surveys. Because the coronavirus S glycoprotein is able to elicit a humoral response which is correlated with the neutralizing antibody response (8, 11–13) and early response to the patients of COVID-19 (14), recombinant coronavirus S ELISAs are usually used (3, 14–16). We found that the N protein is the most appropriate antigen to use for anti-MERS-CoV antibody detection in early serum samples and that use of N and S1 ELISAs in combination can improve the diagnostic sensitivity and specificity (6).
